# Cotton *WATs* Modulate SA Biosynthesis and Local Lignin Deposition Participating in Plant Resistance Against *Verticillium dahliae*

**DOI:** 10.3389/fpls.2019.00526

**Published:** 2019-04-26

**Authors:** Ye Tang, Zhennan Zhang, Yu Lei, Guang Hu, Jianfen Liu, Mengyan Hao, Aimin Chen, Qingzhong Peng, Jiahe Wu

**Affiliations:** ^1^Hunan Provincial Key Laboratory of Plant Resources Conservation and Utilization, College of Biology and Environmental Sciences, Jishou University, Jishou, China; ^2^State Key Laboratory of Plant Genomics, Institute of Microbiology, Chinese Academy of Sciences, Beijing, China; ^3^Key Laboratory for the Creation Cotton Varieties in the Northwest, Ministry of Agriculture, Join Hope Seeds Corporation, Ltd., Changji, China; ^4^Research Base, State Key Laboratory of Cotton Biology, Zhengzhou University, Zhengzhou, China

**Keywords:** *Gossypium hirsutum* L., *Verticillium dahliae*, verticillium wilt, auxin, salicylic acid, lignin

## Abstract

Verticillium wilt, caused by *Verticillium dahliae*, seriously limits cotton production. It is difficult to control this pathogen damage mainly due to the complexity of the molecular mechanism of plant resistance to *V. dahliae*. Here, we identified three homologous cotton *Walls Are Thin* (*WAT*) genes, which were designated as *GhWAT1, GhWAT2*, and *GhWAT3.* The *GhWATs* were predominantly expressed in the roots, internodes, and hypocotyls and induced by infection with *V. dahliae* and treatment with indole-3-acetic acid (IAA) and salicylic acid (SA). *GhWAT1*-, *GhWAT2*-, or *GhWAT3*-silenced plants showed a comparable phenotype and level of resistance with control plants, but simultaneously silenced three *GhWATs* (*GhWAT123*-silenced), inhibited plant growth and increased plant resistance to *V. dahliae*, indicating that these genes were functionally redundant. In the *GhWAT123*-silenced plants, the expression of SA related genes was significantly upregulated compared with the control, resulting in an increase of SA level. Moreover, the histochemical analysis showed that xylem development was inhibited in *GhWAT123*-silenced plants compared with the control. However, lignin deposition increased in the xylem of the *GhWAT123*-silenced plants compared to the control, and there were higher expression levels of lignin synthesis- and lignifications-related genes in the *GhWAT123*-silenced plants. Collectively, the results showed that *GhWATs* in triple-silenced plants acts as negative regulators of plant resistance against *V. dahliae.* The potential mechanism of the WATs functioning in the plant defence can modulate the SA biosynthesis and lignin deposition in the xylem.

## Introduction

Cotton verticillium wilt caused by *Verticillium dahliae* is a soil-borne fungal vascular disease, leading to annual yield losses of more than 30% and a serious economic loss of approximately 250–310 million dollars in China (Gong et al., 2017). However, it is difficult to control the pathogen damage to cotton plants, although many attempts have been made, including attempts with fungicides and cultural approaches (Mohamed and Akladious, 2017; Wei and Yu, 2018). In general, to protect plants from pathogen damage, disease resistance cultivars should be widely planted. Although some genes/proteins have been characterised in cotton plant defence against pathogens, few effective candidate genes have been updated for use in disease resistance breeding (Cai et al., 2009; Zeng et al., 2016; Gong et al., 2017). Therefore, the molecular mechanism of plant resistance to *V. dahliae* and the functional dissection of defence-related genes need to be further explored.

The molecular mechanisms of cotton plant defence against *V. dahliae* are complex. Recently, SA mediated resistance and lignin, as the major physical barriers of cell wall, were reported to play an important role in cotton plant defence against *V. dahliae* (Pieterse et al., 2012; Seyfferth and Tsuda, 2014; Yan and Dong, 2014; Zhu et al., 2018). In SA-related resistance, the SA signalling pathway mainly participates in Arabidopsis plant defence through some hub components, including non-expressor of pathogenesis-related genes 1 (NPR1), NPR3/NPR4, TGAs and pathogenesis-related gene (PR) proteins (Shah, 2003; Fu et al., 2012; Moreau et al., 2012; Seyfferth and Tsuda, 2014; Yan and Dong, 2014; Ding et al., 2018). In cotton, many SA signalling-related genes have been characterised in plant resistance to *V. dahliae* (Chen et al., 2016; Yang et al., 2017; Gong et al., 2018). For instance, the expression of cotton *ribosomal protein L18* (*GaRPL18*) was upregulated by SA treatments, and silencing *GaRPL18* significantly reduced plant resistance to *V. dahlia*e (Gong et al., 2017). The *GhSNAP33* mediates cotton resistance to *V. dahliae* infection by SA treatments (Wang et al., 2018). Additionally, the SA synthesis pathways are also involved in plant defence (Durrant and Dong, 2004; Seyfferth and Tsuda, 2014; Zhou et al., 2018). Such as in Arabidopsis, *isochorismate synthase 1* (*ICS1*) is the major required gene for SA synthesis; *enhanced disease susceptibility 1* (*EDS1*) and *phytoalexin deficient 4* (*PAD4*) positively regulate SA biosynthesis by a feedback loop and the mobilisation of salicylic acid defence (Wildermuth et al., 2001; Rietz et al., 2011). In cotton, ectopic expressions of *GhPGIP1* can upregulate the expression levels of *ICS1, EDS1*, and *PAD4*, enhancing resistance to *Verticillium* (Liu et al., 2017). Cotton *EDS1* can promote SA biosynthesis to enhance plant defence against *V. dahliae* (Su et al., 2014; Zhang et al., 2016, 2017). Together, the SA synthesis and signalling response participate in cotton resistance to *V. dahliae*.

Recently, an increasing number of reports have shown that lignin accumulation in the secondary cell walls of cotton plants enhanced plants sensitivity to *V. dahliae* pathogen infection and colonisation (Xu et al., 2011; Shi et al., 2012; Gao et al., 2013). For example, the ectopic expression of the cotton laccase gene *GhLAC15* in Arabidopsis increased cell wall lignifications, strengthening resistance against *V. dahliae* (Zhang Y. et al., 2018). The lignin contents in different cotton varieties were positively correlated with plant resistance against *V. dahliae*, resulting from increasing lignifications and cross-linking of lignin components (Xu et al., 2011). An ethylene response-related factor *GbERF1-like* gene contributed to the resistance to *V. dahliae* through positively regulating lignin synthesis-related genes and enhancing the lignin content (Guo et al., 2016). Interestingly, *GhUMC1* was identified to be involved in cotton resistance to *V. dahliae* through both the SA signalling pathway and lignin synthesis (Zhu et al., 2018). Additionally, other hosts of *Verticillium* pathogens can increase their resistance through lignin deposition in the secondary cell wall or vascular tissue. For instance, *Verticillium* infection triggers *de novo* xylem formation in Arabidopsis, which depends on NAC domain 7 to maintain plant growth under stress (Reusche et al., 2012). Lignin composition and accumulation in plants contributed to resisting *V. dahliae* colonisation by strengthening the cell walls in *pepper* (Novo et al., 2017). The tomato R gene *Ve* participated in the plant resistance response to *V. dahliae* by increasing lignin- and PAL-related gene expression (Gayoso et al., 2010). Together, these studies documented that SA and lignin play important roles in plant defence against *Verticillium* pathogen infection. However, some novel SA- and/or lignin-related genes remain to be mined for cotton disease resistance breeding.

Arabidopsis *walls are thin 1* (*WAT1*), which is a *Medicago truncatula NODULIN 21* (MtN21) homologue gene, was identified as a member of the drug/metabolite transporter (DMT) superfamily (Gamas et al., 1996; Ranocha et al., 2010). WAT1 functions in the regulation of auxin polar transport, secondary cell wall deposition and cell elongation (Ranocha et al., 2010). WAT1 is localised in the tonoplast and mediates auxin transport across the tonoplast from the vacuole to external space, regulating the homeostasis of auxin intracellular metabolism (Ranocha et al., 2010, 2011, 2013). Recent reports have shown that WAT1 participates in plant broad-spectrum resistance to vascular pathogens by regulating SA metabolism and signalling transduction through affecting auxin polar transport (Ranocha et al., 2010, 2013; Denancé et al., 2013). Thus, WAT1 participates in plant defence against vascular pathogens, possibly involved in SA synthesis and secondary cell wall lignifications.

In this study, we identified three cotton *WAT* genes, *GhWAT1, GhWAT2*, and *GhWAT3*, and dissected their function in plant defence against *V. dahliae*. The knockdown of three *GhWATs* by a virus-induced gene silencing (VIGS) method can increase plant resistance. According to molecular, biochemical and histological analyses, the results showed that simultaneously silencing three *GhWATs* promoted the expression of SA synthesis-related genes and increase SA level. However, the knockdown of *GhWATs* increases lignin deposition in the xylem sections. Collectively, the data showed that *GhWATs* as negative regulators increases disease resistance in silenced-plants infected by *V. dahliae*, possibly resulting from modulating SA synthesis and lignin accumulation.

## Materials and Methods

### Plant Materials, Growth Conditions, and Hormone Treatment

*G. hirsutum* cv. BD18 seeds were soaked in tap water at 37°C overnight, and then the imbibed seeds were placed on wet tissue to germinate in the dark at 28°C for 2 days. Similar germinating seeds were transferred into a sterilised soil mixture (a complex of organic matter soil and vermiculite) or to hydroponic conditions (Hoagland nutrient solution) in a growth chamber at 25°C with a 16/8 h light/dark photoperiod and 75% relative humidity.

*N. benthamiana* was cultivated in a sterilised soil mixture with the same conditions as described for *G. hirsutum* growth for the subcellular location analysis.

To analyse the gene response to phytohormones, 3-week-old seedlings were transferred to Hoagland nutrient solution. IAA or SA was added into culture solution. IAA and SA were adjusted to 50 μM and 2 mM, respectively (Shi et al., 2010; Hu et al., 2018). The seedlings were mock-treated with the corresponding concentration of ethanol at the same time. Then roots were harvested for RNA extraction at 0, 0.25, 0.5, 1, 4, 6, and 12 h after hormone and mock treatment. Over 10 seedlings were collected at each time point. The experiments were performed with three biological replicates.

### Phylogenetic Analysis

The three homologous GhWATs were retrieved in the Cottongen database^1^. The functional domains of GhWATs and the transmembrane helices were predicted using UniProt database^2^. Protein hydrophobicities analyses were performed by DNAMAN 7.0. BlastP was employed to analyse GhWATs homologous sequences in NCBI^3^. The amino acid sequence alignment and phylogenetic relationship analyses were performed on ClustalX 2.1 and MEGA5.0, respectively.

### VIGS Mediated by *Agrobacterium tumefaciens*

Virus-induced gene silencing technology has been successfully reported to determine cotton gene function (Gao et al., 2013; Pang et al., 2013). Specific fragments of the *GhWAT1, GhWAT2, GhWAT3* and positive control *GhPDS* genes were amplified by PCR, were digested with *Bam*H I and *Kpn* I and then cloned into the tobacco rattle virus (TRV) vector pYL156, generating pYLW1, pYLW2, pYLW3, and pYLPDS vectors, respectively. In addition, we cloned shorter fragments of *GhWAT1, GhWAT2*, and *GhWAT3* by PCR with corresponding specific primers, and these were tandemly inserted into pYL156 to form the pYLW123 vector. Then, all pYL-candidate gene vectors and the auxiliary vector pYL192 were transformed into *A. tumefaciens* strains GV3101 by electroporation. *A. tumefaciens* containing the indicated vectors was cultured in LB medium with 50 μg/mL kanamycin and 40 μg/mL rifampicin at 28°C overnight. The Agrobacterium cells were collected and then resuspended in MMA solution (10 mM *N*-morpholino ethanesulfonic acid, 10 mM MgCl_2_, and 200 mM acetosyringone). Every suspension concentration was adjusted to a 1.2 value of OD_600_. The Agrobacterium cells containing the aforementioned vectors were equally mixed with those containing pYL192 and were incubated at room temperature for 3 h in the dark. Finally, the mixed Agrobacterium cells were injected into the fully expanded cotyledons of 7-day-old seedlings by a 1-mL needleless syringe. The experiment was performed three times. Each sample comprised of more than 50 cotyledons. The primers used in the experiment are shown in Supplementary Table S1.

### Pathogen Infection and Plant Disease Assays

The highly aggressive defoliating fungal pathogen *V. dahliae* strain V991 was provided by the Institute of Plant Protection, CAAS, Beijing, China. And then it was cultured on potato dextrose agar plates for 3 days at 25°C in the dark. Then, hyphae were transferred into Czapek’s medium (NaNO_3_, 0.3% w/v; MgSO_4_, 0.1% w/v; KH_2_PO_4_, 0.1% w/v; FeSO_4_, 0.0002% w/v; KCl, 0.1% w/v; and sucrose, 3% w/v; pH 6.0) for harvesting the fungal conidia. Plants were inoculated with *V. dahliae* by the root dip method previously described (Zhang et al., 2012). In brief, the 3-week-old plants were dipped into a fungal conidia suspension (1 × 10^6^ conidia/mL) for 1 min and transferred into a growth chamber with soil culture to allow the disease to progress. The conditions of cultivation were consistent with the plants’ growth conditions. To investigate the disease resistance of silenced plants to *V. dahliae*, the disease index (DI), fungal recovery assay and fungal biomass quantification assay were performed. The DI was calculated according to the method reported by Wang et al. (2004) with minor modification. In brief, the degree of plant infection by the fungi was divided into four grades according to disease scores ranging from 1 and 4 (1: healthy plants, or <10% of the leaves show yellowing or yellow spots; 2: 10–<40% of the leaves show yellow spots, a few leaves fall off; 3: 40–<70% of the leaves show yellow or brown spots, some leaves fall off; 4: >70% of the leaves showed brown spots, many leaves fall off, even the death of the whole plant). The DI was calculated following formulae: DI = [(Σ disease grade × number of infected plants)/total checked plants × 4] × 100%. The fungal recovery experiment was performed according to the method previously described by Fradin et al. (2009). The fungal biomass quantification assay was performed according to the method described by Wang et al. (2017). The *β-tubulin* gene of *V. dahliae* was used to measure the fungal biomass by qPCR. *GhUB-7* was used to normalise the DNA quantity of the plant. The same experiments were performed with three biological replicates.

### DNA/RNA Extraction and qRT-PCR

For fungal growth biomass, fungal DNA was extracted using five cotton stems through the CTAB method at 3 weeks after *V. dahliae* infection (Muhammad et al., 2013). Total RNA from cotton samples, including the roots, internodes, leaves, cotyledons, and hypocotyls, was extracted using a plant total RNA extraction kit (Sangon, Shanghai, China) following the manufacturer’s protocol. First-strand cDNA was synthesised from 3 μg of total RNA using the Goldenstar RT6 cDNA Synthesis kit Ver. 2 (Tsingke, Beijing, China) to analyse the expression levels of related genes by qRT-PCR. Quantitative detection was performed using the 2 × T5 Fast qPCR mix kit (Tsingke, Beijing, China) in a real-time quantitative PCR instrument (Bio-Rad, Foster City, CA, United States) and the relative quantification was calculated using the comparative Ct method (Livak and Schmittgen, 2001; Li et al., 2005). To normalise gene expression, *GhUB-7* was used as an internal control. The experiments were performed with three technical replicate and three biological replicates (*n* ≥ 3). All primers used in the experiments are shown in Supplementary Table S1.

### Subcellular Location

The coding sequences of *GhWAT1, GhWAT2*, and *GhWAT3* were amplified from *G*. *hirsutum* cDNA with the following specific primer pairs: WAT1-F/R, WAT2-F/R, and WAT3-F/R, respectively. The tonoplast marker gene *AtTIP_2_* was amplified with TIP_2_-F/R primer pairs from Arabidopsis cDNA. The pUC-mGFP4 and pUC-mCherry vectors were double digested with *Kpn* I and *Xba* I (TAKARA, Dalian, China). Then, the amplified products of *GhWATs* and *AtTIP_2_* were inserted into the pUC-mGFP4 and pUC-mCherry vectors, respectively, by using a homologous recombination kit (Vazyme, Nanjing, China) according to the manufacturer’s guidelines, generating the GhWATs:GFP and AtTIP_2_:mCherry vectors. The primers used in PCR are shown in Supplementary Table S1.

To observe the subcellular location of GhWATs, the aforementioned plasmids were extracted using the QIAGEN Plasmid Midi Kit (QIAGEN, Germany). The *N. benthamiana* mesophyll protoplasts were isolated and transformed according to methods reported previously (Schweiger and Schwenkert, 2014; Lei et al., 2015). Then, protoplasts that had been incubated for 12–18 h at room temperature under a low level of light were observed under a confocal laser scanning microscope (TCS SP2; Leica, Germany).

### Histochemical Staining and Lignin Content Analysis

Histochemical staining with Wiesner reagent was used to visualise xylem development and lignin deposition (Xu et al., 2011). More than five cotton internodes and hypocotyls of the *GhWAT123*-silenced and control plants were crosscut into sections by hand and directly dipped in phloroglucinol solution (2% phloroglucinol dissolved in 95% ethanol) to incubate for 10 min, followed by treatment with 18% HCl for 20 min. The stained sections were observed and photographed using a stereomicroscope (DM2500; Leica, Germany). The lignin contents of hypocotyls from the silenced and control plants were determined by the Klason method as previously described (Ronald et al., 1994; Abdullah et al., 2006). The same experiment was conducted with three biological replicates, and each replicate comprised more than five individual seedlings.

### Paraffin Sectioning

To observe the xylem vessels of the different samples, a paraffin sectioning was carried out according to the method described by Kong et al. (2017) with minor modifications. The five young stems from the silenced and control plants were fixed with FAA fixation solution (95% ethanol, 5% glacial acetic acid, 37% formalin and DDW) for 24 h. The paraffin sections were stained with safranin solution (1% safranin dissolved in 50% ethanol) overnight, which stains lignified tissues, such as the xylem. The stained paraffin sections were rinsed with 95% ethanol and observed using an inverted microscope (Axiovert 200 M; Zeiss, Germany) (Vazquez-Cooz and Meyer, 2002; De Micco and Aronne, 2007; Bond et al., 2008).

### SA Extraction and Measurement

The SA extraction was performed as described by Sun et al. (2014). In brief, 100–200 mg root samples were ground to powder in liquid nitrogen and homogenised twice with the cold extraction buffer (methanol:ddH_2_O:glacial acetic acid = 80:19:1) for oscillation overnight in the dark at 4°C. The extraction was evaporated dry with N_2_. The dry powder was dissolved with 300 μL methanols; and the solution was filtered by 0.22 μm filters. SA content was measured by HPLC-MS/MS performed by AB SCIEX QTRAP 4500 system (AB SCIEX, Foster City, CA, United States) as described by Zhou et al. (2018). The experiment was repeated three times, with samples containing more than three seedlings.

## Results

### Identification and Characterisation of *G. hirsutum* WAT1

**FIGURE 1:**
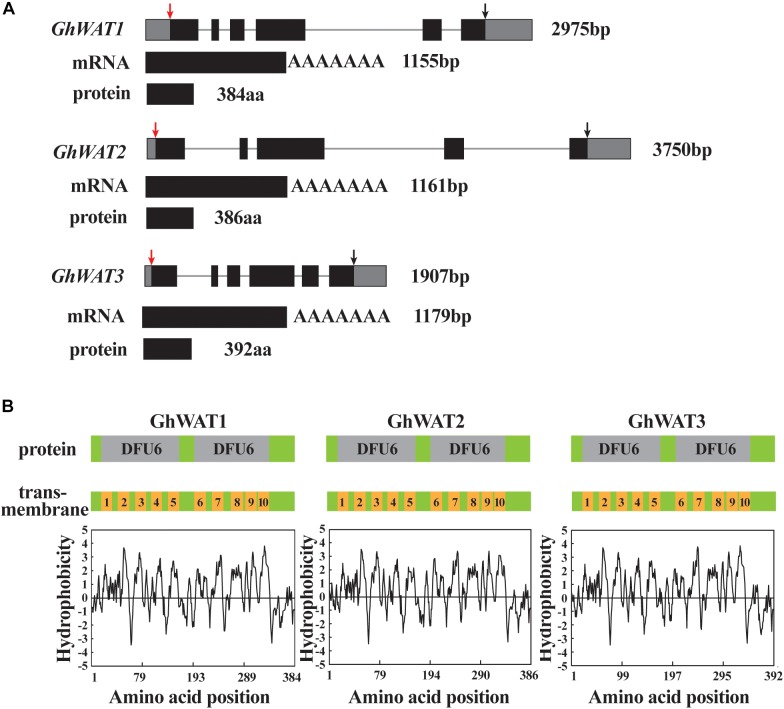
Three *GhWATs* genes structural characteristics and their corresponding predicted proteins. **(A)** *GhWATs* gene structure, mRNA and deduced proteins. Lines represent introns, black boxes represent exons in gene structure. The red arrow points to ATG and the black points to stop codon. **(B)** The protein domains and transmembrane domains of *GhWATs* according to predictions of hydrophobicity regions. The GhWATs contain two DUF6 domains; each of the DFU6 domains encompasses five transmembrane-spanning domains.

Recently, reports have shown that many genes associated with lignin deposition in the cell wall and xylem vessels participate in cotton plant defence against *V. dahliae* (Xu et al., 2011; Gao et al., 2013). Arabidopsis WAT1 functions in secondary wall formation in interfascicular and xylary fibres, which participates in plant resistance to *Ralstonia solanacearum, V. dahliae*, and other vascular pathogens (Denancé et al., 2013, 2014). Thus, we were prompted to explore cotton WAT functions in plant defence and lignin synthesis. Three homologous WATs in *G. hirsutum* were identified by blasting Arabidopsis WAT1 in the available genomic sequence of *G. hirsutum*^4^, and they were designated *GhWAT1* (Gh_A05G1621 and Gh_D05G1805 located in the A and D subgenomes, respectively), *GhWAT2* (Gh_A06G1836), and *GhWAT3* (Gh_A01G0922 and Gh_D01G0964). Both *GhWAT1 and GhWAT3* genes contain six exons and five introns, while *GhWAT2* only has five exons and four introns. *GhWAT1, GhWAT2*, and *GhWAT3* encode 384, 386, and 392 amino acid peptides, respectively (Figure 1A). They encode proteins belonging to the EamA-like transporter (DOMAINS OF UNKNOWN FUNCTION 6, DUF6) family (Figure 1B). According to the WAT hydrophobicity profile analysis, two DUF6s possess ten potential alpha helix transmembrane-spanning domains (Figure 1B), which is consistent with the structure of Arabidopsis WAT1 (Ranocha et al., 2010). Amino acid sequence alignment analysis suggested that GhWATs exhibited sequence highly identifies with Arabidopsis WAT1 and *Medicago sativa* N21, reaching 88.4 and 88.8%, respectively. The protein similarities of the three GhWATs reached 92.9%, indicating that there could be functional redundancy among them (Supplementary Figure S1).

### Analyses of *GhWAT* Expression Profiles

**FIGURE 2:**
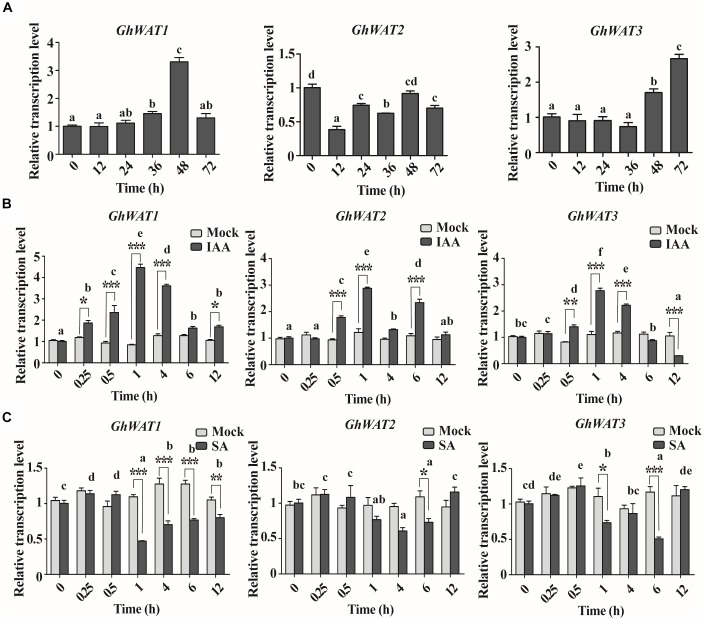
The pathogen and hormone-induced expression patterns of three *GhWATs*. **(A)** The expression analysis of *GhWAT1, GhWAT2*, and *GhWAT3* in cotton roots in response to *V. dahliae* infection. Relative expression levels were normalised to the levels at 0 h of infection. *GhUB-7* was used as a reference gene. The error bars represent the standard error of the mean (SEM) of three biological replicates. Different letters represent statistically different means at *P* < 0.05 (one-way ANOVA with a Duncan *post hoc* test). **(B,C)** The expression levels of three *GhWATs* in plants treated with 50 μm IAA **(B)**, 2 mM SA **(C)** and the corresponding mock treatments. Relative expression levels were normalised to the levels at 0 h of infection. *GhUB-7* was used as a reference gene. The error bars represent the standard error of the mean (SEM) of three biological replicates. As compared with the corresponding time of the mock treatment, two-way ANOVA with a Bonferroni *post*-test: ^∗^*P* < 0.05, ^∗∗^*P* < 0.01, ^∗∗∗^*P* < 0.001. Comparing all the time points, different letters represent statistically different means at *P* < 0.05 (two-way ANOVA followed by Duncan *post hoc* test).

To investigate the expression pattern of *GhWAT1, GhWAT2*, and *GhWAT3* in plant tissues, the *GhWAT* mRNA levels of samples from roots, internodes, leaves, cotyledons, and hypocotyls were analysed by qPCR. As shown in Supplementary Figure S2, *GhWAT1, GhWAT2*, and *GhWAT3* were predominantly expressed in the roots, internodes, and hypocotyls but were slightly expressed in the leaves and cotyledons. Since Arabidopsis *WAT1* participates in plant defence through regulating SA synthesis and IAA transport (Denancé et al., 2013), we evaluated the *GhWATs* expression levels of plants infected with *V. dahliae* and treated with either IAA or SA. The results showed that the expression levels of *GhWAT1* exhibited an increasing trend over 0–72 h ranges in the treated roots (0 h-treated samples collected from the parallel plants without any treatment as a control) and, specifically, that at 48 h the expression level significantly increased compared with other time points. The *GhWAT2* expression levels showed significantly decreased at 12 and 36 h among all time points. *GhWAT3* expression was significantly upregulated at 48 and 72 h in the treated roots among all time points. These data suggested that the three homologous *GhWATs* had different expression responses to *V. dahliae* infection (Figure 2A).

The IAA treatment was first performed to investigate *GhWATs* expression profiles in hormone-induced plants. As shown in Figure 2B, *GhWAT1* exhibited significantly upregulated expression in 0.25, 0.5, 1, 4, and 12 h-treated roots compared with the mock treatment; among all time points, the *GhWAT1* at 0.5, 1, and 4 h were significantly upregulated expression. The expression level of *GhWAT2* was significantly upregulated in 0.5, 1, and 6 h-treated roots compared with the mock treatment, and the three time points showed significant upregulation in *GhWAT2* expression among all time points. The *GhWAT3* expression levels significantly increased in 0.5, 1, and 4 h-treated roots and then significantly decreased at the 12 h-treated samples compared with the mock treatment; the expression levels of *GhWAT3* were significantly upregulated at 0.5, 1, and 4 h among all time. Then, *GhWATs* expression levels were checked under SA treatment. The Compared with mock treatment samples, expression levels of *GhWAT1* significantly decreased in 1, 4, 6, and 12 h samples, those of *GhWAT2* significantly downregulated in 6 h samples and *GhWAT3* significantly decreased expression in 1 and 6 h samples. Among all the time points, the expression of *GhWAT1, GhWAT2*, and *GhWAT3* at various time points showed significant differences (Figure 2C). These results suggested that the expression of the three *GhWATs* was induced by IAA treatments and repressed by SA treatments.

### The Knockdown of *GhWATs* Increases Plant Resistance to *V. dahliae*

**FIGURE 3:**
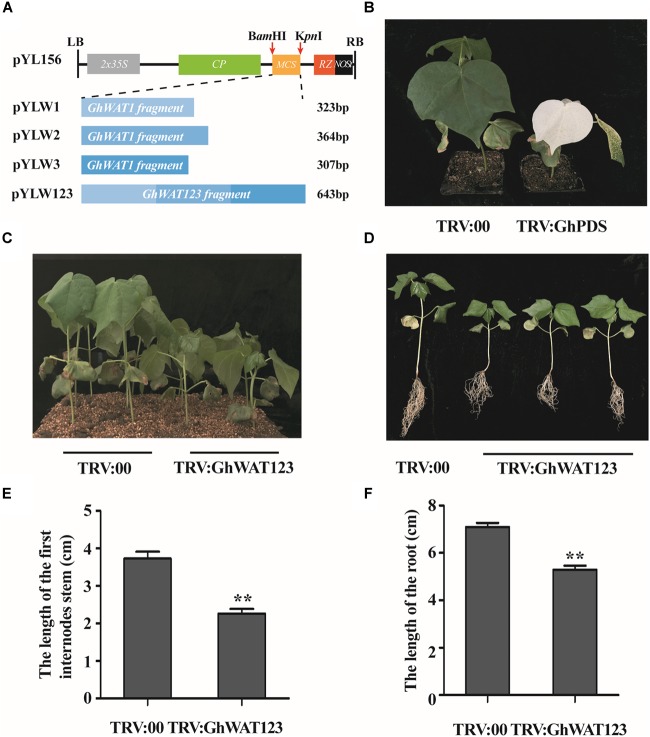
Development of *GhWATs*-silenced plants and their phenotypes by VIGS. **(A)** Schematic diagram of the pYL156, pYLW1, pYLW2, pYLW3, and pYLW123 vectors. LB and RB, left and right borders of T-DNA; 2 × 35S, CaMV35S promoter with the duplicated enhancers; CP, coat protein; MCS, multiple cloning sites; RZ, self-cleaving ribozyme; Nos, NOS terminator. **(B)** Photobleaching phenotypes of *GhPDS*-silenced plant leaves (positive control) at 2 weeks post-Agro-infiltration. **(C,D)** Phenotypic characteristics of *GhWAT123*-silenced and control plants grown in plots with soil and exposed to Hoagland nutrient solution. **(E,F)** The length of the first internode **(E)** and the root length **(F)** of silenced plants and the control plants. TRV:00 is control plants injected with the pYL156 vector. Statistical significance was determined by Student’s *t*-test: ^∗∗^*P* < 0.01. The error bars represent the SEM of three biological replicates.

To characterise the function of GhWATs in plant defence, the VIGS method was used to generate *GhWAT1*-, *GhWAT2*-, and *GhWAT3-*silenced plants. And the vectors of pYL156, pYLW1, pYLW2, pYLW3, and pYLW123 construction showed in Figure 3A. Seven-day-old plants were injected with *A. tumefaciens* strain GV3101 cells containing the indicated vectors using a 1-mL syringe without a needle. At 14 days post-Agro-infiltration, the positive control plants that contained silenced *phytoene dehydrogenase* (*GhPDS*) genes showed white bleached leaves (Figure 3B), which indicated that the TRV-VIGS system worked well in cotton plants. At this time point, the corresponding *GhWATs* expression levels of the silenced plant leaves and roots were detected by qPCR to analyse the silencing efficiency. As shown in Supplementary Figure S3, the expression levels of *GhWAT1, GhWAT2*, and *GhWAT3* significantly decreased in the silenced leaves and roots compared with the those in the control plants injected with the pYL156 vector (as the control named TRV:00), and the expression levels of *GhWAT1, GhWAT2*, and *GhWAT3* reduced, exhibiting 0.13-, 0.35-, and 0.17-fold of the control in leaves and 0.3-, 0.04-, and 0.09-fold in roots, respectively. Expectedly, the expression of *GhWAT1, GhWAT2*, and *GhWAT3* was greatly reduced in the *GhWAT123*-silenced plants (Supplementary Figure S3). At 21 days post-Agro-infiltration, the *GhWAT1*-, *GhWAT2*-, and *GhWAT3*-silenced plants showed comparable phenotypes with the control plants, while the *GhWAT123-*silenced plants exhibited smaller phenotypes than control plants with significantly lower biomass (Supplementary Figures S4A,B). As shown in Figure 3C,D, the plant height and root length of the *GhWAT123*-silenced plants were clearly shorter than those in the control. The first internode growth in the *GhWAT123*-silenced plants was significantly inhibited compared with that in the control, with the length reduced by 39% of that of the control (Figure 3E). Similar results were detected for the root growth of the *GhWAT123-*silenced plants, which showed a reduction of 25% of that of the control (Figure 3F).

**FIGURE 4:**
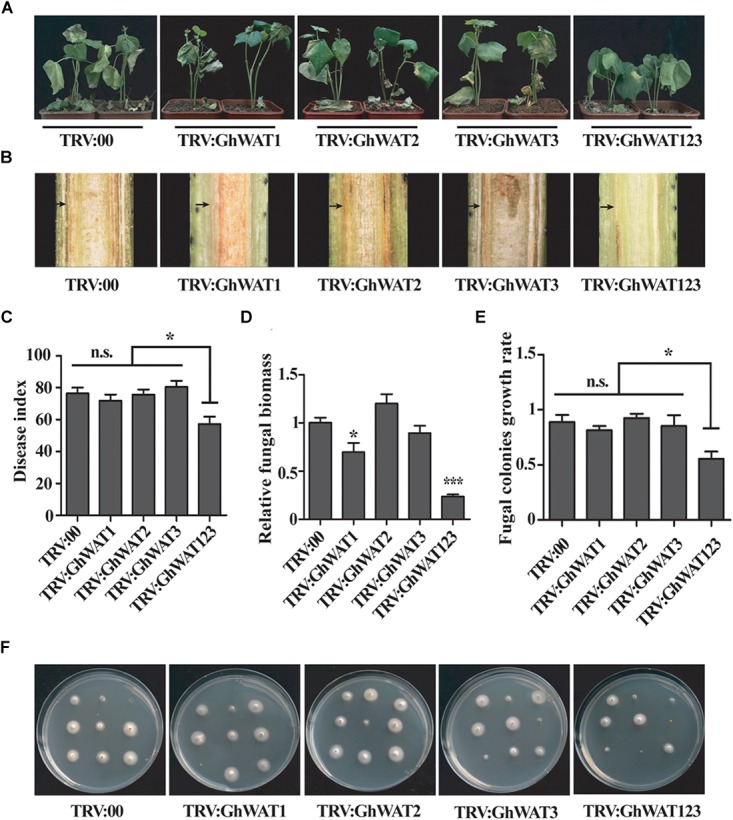
The simultaneous knockdown of three *GhWATs* increased plant resistance to *V. dahliae*. **(A)** Disease symptoms of the three single *GhWAT-*silenced plants, the *GhWAT123*-silenced plants and the control plants inoculated with *V. dahliae* (10^6^ conidia/mL) at 21 days. **(B)** The browning phenotypes in the xylem of longitudinal cross-sections of silenced hypocotyls at 21 days after infection. The black arrow points to the browning xylem. **(C)** The disease index of the control, three single *GhWATs*- and *GhWAT123*-silenced plants 21 days after inoculation. The star represents *P* < 0.05 (one-way ANOVA with a Duncan *post hoc* test). n.s. = 0.5536. **(D)** The relative *V. dahliae* fungal biomass in the infected stems was quantified by qRT-PCR. The DNA contents of the *V. dahliae β-tubulin* gene in the *V. dahliae-*inoculated plants were analysed by qPCR. The *GhUB*-*7* DNA levels were normalised to the pathogen DNA contents of the *β-tubulin* gene. The statistically significant differences compared with the *GhWAT123*-silenced plants were determined by Student’s *t*-test (^∗^*P* < 0.05, ^∗∗∗^*P* < 0.001). Student’s *t*-test was used to determine significant differences between the TRV:00 control and other silenced plants. TRV:00 is injected with the pYL156 vector. Error bars represent the SEM of three biological replicates. **(E)** Statistics of the fungal colonies. The number of fungal colonies of each plate means the rate of recovery growth rate. The star represents *P* < 0.05 (one-way ANOVA with a Duncan *post hoc* test). n.s. = 0.3942. **(F)** A fungal recovery assay. The first internode of the plants inoculated with *V. dahliae* for 21 days was cultured on potato dextrose agar medium for 3 days.

Previous reports showed that Arabidopsis *WAT1* mutation conferred broad-spectrum resistance to vascular pathogens, such as *V. dahliae* (Denancé et al., 2013). The *GhWAT1-, GhWAT2*-, *GhWAT3*-, and, *GhWAT123-*silenced plants and the control plants were inoculated with *V. dahliae* through the root dip method. Three weeks after inoculation, the single-silenced plants and the control showed a similarly high level of susceptibility to the pathogen, with disease symptoms including plant wilt, stunted growth, leaf chlorosis and even defoliation (Figure 4A). However, the triple-silenced plant, *GhWAT123-*silenced plant, showed a stronger level of resistance to *V. dahliae* compared with the single-silenced plant and the control (Figure 4A). As observed in the corresponding longitudinal stem sections, there were darker brown vascular vessels in the single-silenced plants and the control plants than in the *GhWAT123-*silenced plants (Figure 4B). The disease index of the *GhWAT123-*silenced plants was significantly reduced compared with the *GhWAT1-, GhWAT2*-, and, *GhWAT3*-silenced plants and the control, which showed decreases of 14.6, 18.4, 23.1, and 19.1%, respectively (Figure 4C). The fungal biomass results in the infected stems showed that the relative fungal DNA levels of the three single *GhWAT*-silenced plants were comparable with the controls, while that of the *GhWAT123-*silenced plants was significantly lower than those of the controls (Figure 4D). To further investigate the effects of *GhWATs* on plant resistance, hyphae recovery growth from cutting sections of the infected stem was assayed. We found that most of the stem sections from the single-silenced plants and the control could grow mycelia, while significantly fewer fungal colonies were observed in the cutting sections from the *GhWAT123-*silenced plants than in those of the other plants (Figure 4E,F). Altogether, these results showed that *GhWAT1, GhWAT2*, and, *GhWAT3* are functionally redundant and together participate in plant defence against *V. dahliae*.

### GhWATs Subcellular Localisation and the Expression Response of Auxin-Related Genes

**FIGURE 5:**
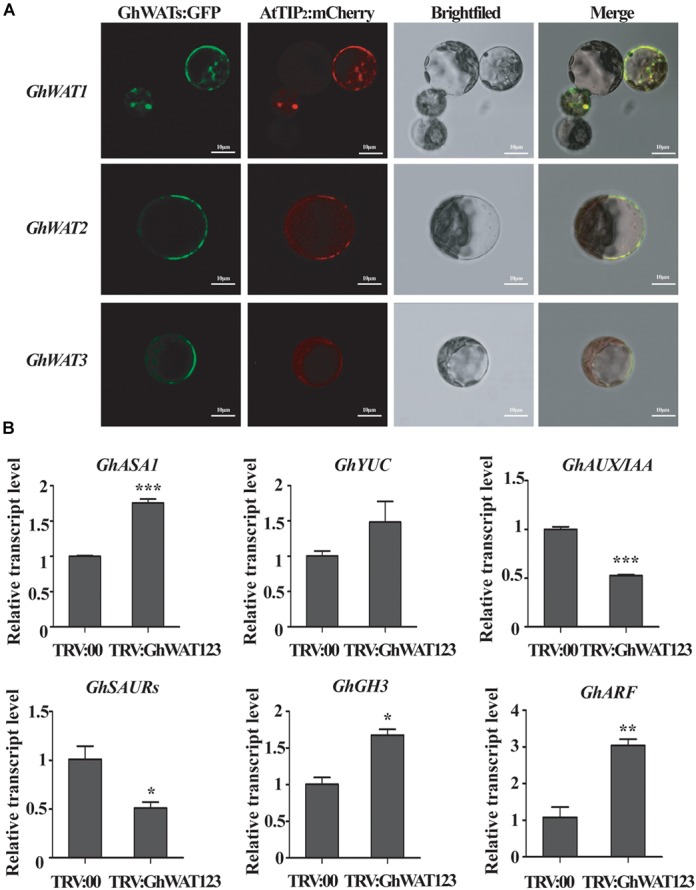
Subcellular location of GhWATs and the expression levels of auxin-related genes. **(A)** The three GhWATs:GFP fusion proteins co-localised with AtTIP2:mCherry in the tonoplast in *N. benthamiana* mesophyll protoplasts. AtTIP2 is a tonoplast protein maker. Scale bars: 10 μm. **(B)** The expression levels of auxin-related genes. TRV:00 is control plants injected with the pYL156 vector, which relative expression level was normalised to ‘1’, and *GhUB-7* was used as a reference gene. Student’s *t*-test were performed in the same experiments (^∗^*P* < 0.05, ^∗∗^*P* < 0.01, ^∗∗∗^*P* < 0.001). Error bars represent the SEM of three biological replicates.

To determine the subcellular localisation of the GhWATs to characterise their function, three vectors containing the GhWATs:mGFP4 fusion protein driven by the CaMV35S promoter were constructed and transiently expressed in *Nicotiana benthamiana* mesophyll protoplasts. A tonoplast protein AtTIP_2_ (AT3G26520):mCherry vector was constructed for use as a co-localised tonoplast maker. When GhWATs:mGFP4 and AtTIP_2_:mCherry were co-expressed in protoplasts, the GFP signal of GhWATs:mGFP4 was merged with the image of AtTIP_2_:mCherry, indicating that the three GhWATs probably localised in the tonoplasts (Figure 5A).

In *wat1* mutant roots, indole metabolism was repressed, and the expression of IAA-related genes was reprogrammed (Denancé et al., 2013; Ranocha et al., 2013). In our studies, the expression levels of two auxin biosynthesis-related genes, *anthranilate synthase α1* (*ASA1*) and *Flavin Monooxygenase-Like Enzyme YUCCA1* (*YUC1*), were increased in the silenced plants compared with the control plants (Figure 5B). The expression levels of four auxin signalling response-related genes, auxin/indole-3-acetic acid (*AUX/IAA*) and *small auxin-up RNAs* (*SAUR*), were significantly downregulated than the control plants, while the expression levels of *auxin-responsive Gretchen Hagen 3* (*GH3*) and *AUXIN RESPONSE FACTOR* (*ARF*) were significantly upregulated (Figure 5B). These data suggested that the knockdown of *GhWATs* affected auxin biosynthesis and signalling response possibly by disrupting auxin polar transport across the tonoplast.

### *GhWATs* Participate in Regulation of SA Synthesis- and Signal Response-Related Genes

**FIGURE 6:**
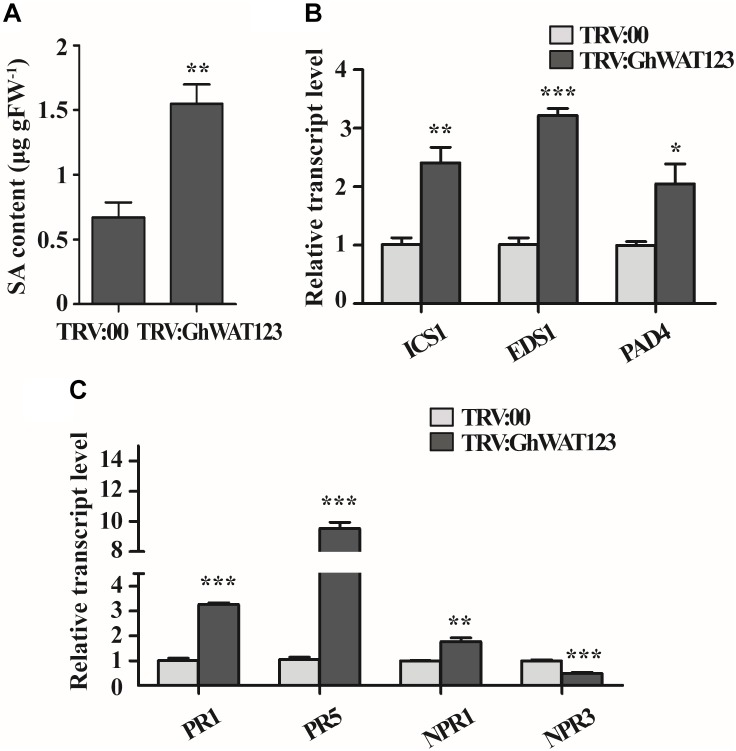
SA contents and expression patterns of SA-related genes. **(A)** SA content in the root of *GhWAT123*-silenced and control plants. FW, fresh weight. **(B)** The expression levels of three SA biosynthesis-related genes, *ICS1, EDS1*, and *PAD4*, in *GhWAT123*-silenced and control plants. **(C)** The expression levels of *PR1, PR5, NPR1*, and *NPR3* in *GhWAT123*-silenced and control plants. TRV:00 is controlled plants injected with the pYL156 vector and the relative expression levels were normalised to ‘1’. *GhUB-7* was used as the reference gene. The statistically significant differences were run by Student’s *t*-test (^∗^*P* < 0.05, ^∗∗^*P* < 0.01, ^∗∗∗^*P* < 0.001). Error bars represent the SEM of three technical replicates.

In both IAA and SA biosynthesis, chorismate acted is shared as a common substrate (Jones and Dangl, 2006; Vlot et al., 2009; Ranocha et al., 2010, 2013; Denancé et al., 2013; Fu and Dong, 2013). Thereby, the SA content in the roots was measured to evaluate the GhWATs role in SA biosynthesis. The SA content in triple-silenced plants was significantly higher than the control (Figure 6A). The expression of *ICS1*, SA synthesis-required gene, was twofold higher in the G*hWAT123*-silenced plants than in the control plants. The expression levels of *EDS1* and *PAD4*, which contribute to the positive feedback loop involved in SA biosynthesis, were significantly increased in the *GhWAT123*-silenced plants compared with the control plants (Figure 6). These results indicated that the knockdown of *GhWATs* can activate SA synthesis and positive feedback loop process, which increases plant resistance to pathogens.

To investigate whether the activation of SA synthesis- and positive feedback loop-related genes could increase the expression of defence-related genes, four genes, including SA signal component factors and defence-related genes, were monitored at the transcript level (Denancé et al., 2013). As shown in Figure 6C, the expression level of *NPR1* in the *GhWAT123*-silenced plants was significantly higher than that in the control, while that of *NPR3* was lower. *PR1* and *PR5* expression was upregulated in the *GhWATs* knockdown plants compared with the control and were 3.2- and 9-fold higher than control plants, respectively. These results showed that the knockdown of *GhWATs* can reprogram the transcription of SA defence-related genes.

### Knockdown of *GhWATs* Inhibits Xylem Development and Locally Increases Lignin Deposition

**FIGURE 7:**
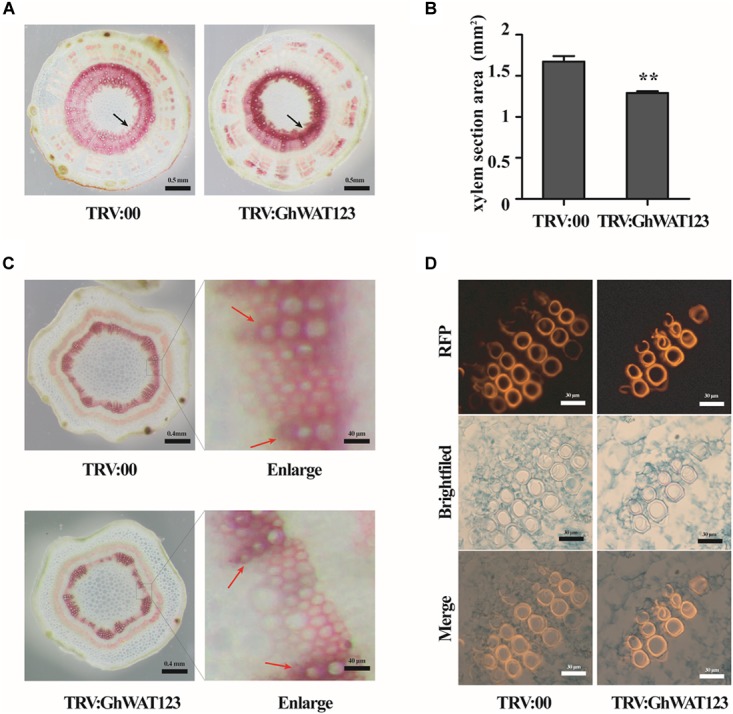
Knockdown of *GhWAT123* inhibited xylem development and locally increased lignin deposition. **(A)** Phloroglucinol-HCl staining of lignin in hypocotyl cross-sections of *GhWAT123*-silenced and control plants. The xylem sections with lignin were stained red. Scale bars represent 0.5 mm. The black arrow points to the xylem of stained red. **(B)** The area of the hypocotyl xylem section of silenced and control plants. The xylem section area measurement was performed using Adobe Photoshop CS5. The mean and SEM came from three biological replicates. Double asterisks indicate statistically significant differences compared with the control, which was determined by Student’s *t*-test (^∗∗^*P* < 0.01). **(C)** Histochemical analysis of transverse sections of young internodes in *GhWAT123*-silenced and control plants. The lignified tissue and interfascicular fibre were stained red using phloroglucinol-HCl. The red arrow points to the xylary fibres, between the red arrows were interfascicular fibres. Scale bars represent 0.4 mm or 40 μm. The right panels were enlarged from the control or silenced plants by approximately 100-fold. **(D)** Safranin staining of the paraffin sections of the control and silenced plants. Scale bars represent 30 μm. TRV:00 is control plants. The same experiment was performed three times.

In Arabidopsis, *WAT1* is required for secondary cell wall deposition and participates in plant resistance to vascular pathogens (Ranocha et al., 2010; Denancé et al., 2013). To investigate secondary cell wall deposition in the *GhWATs* knockdown plants, cross-sections of hypocotyls stained with phloroglucinol-HCl were observed. The xylem in the vascular tissue showed a red colour with Wiesner reagents, which stain lignin. As shown in Figure 7A,B, compared with the xylem area of the control (1.27 mm^2^), the xylem area of the *GhWAT123*-silenced hypocotyls (1.65 mm^2^) was significantly smaller. However, the red colour density of the xylem area was darker compare with the control. The lignin contents of whole hypocotyls in the silenced plants, which were analysed by the Klason method, were slightly increased compared with those in the control (Supplementary Figure S5A), resulting in lignin deposition was higher in the smaller xylem area of *GhWAT123*-silenced plants than control. The expression of lignin synthesis-related genes, including *Phe:phenylalanine ammonia-lyase* (*GhPAL*), *cinnamate 4-hydroxylase* (*GhC4H*), *4-coumarate:CoA ligase* (*Gh4CL*), *p-coumarate 3-hydroxylase* (*GhC3H*), *Caffeoyl-CoA O-methyltransferase* (*GhCCoAMT*), *cinnamyl alcohol dehydrogenase* (*GhCAD*), and *cinnamoyl-CoA reductase* (*GhCCR*), was upregulated in the *GhWAT123*-silenced plants compared with the control (Supplementary Figure S5C). Then the expression levels of three genes involved in the last steps in lignification, *laccase 1* (*GhLac1*), *peroxidase 1* (*Ghpox1*), *dirigent 1* (*GhDIR1*), were detected by qPCR. The expression of *GhLac1* was significantly upregulated in silenced-plants compared with the control (Supplementary Figure S5B). These results indicated that silencing *GhWATs* can repress xylem development but increase lignin accumulation in xylem sections.

To further evaluate *GhWATs* function in xylem development, transverse sections of young internodes were stained with phloroglucinol-HCl. The xylem in silenced internodes was stained a slightly purple colour, indicating that the lignin content in the xylem was higher in the *GhWATs* knockdown internodes than in the control internodes (Figure 7C in enlarging). And the secondary cell wall thickness of interfascicular fibres decreased in the *GhWAT123*-silenced plants compared with the control plants (Figure 7C). To further observe the xylem structure, paraffin cross-sections were analysed. Safranin was used to stain the lignified tissues to identify lignin-rich cell walls, which exhibited a fluorescent red or orange colour (Vazquez-Cooz and Meyer, 2002; De Micco and Aronne, 2007; Bond et al., 2008). As shown in Figure 7D, a string of xylem vessels in the silenced internodes contained almost two to three layers, while that in the control internodes had three to four layers. The fluorescence of the vessels was stronger in the silenced plants than in the control plants, indicating that more lignin was deposited around xylem vessels in the silenced plants than in the control plants. These data confirmed that the knockdown of *GhWATs* represses xylem development and locally increases lignin deposition in xylem sections.

## Discussion

Verticillium wilt causes devastation to cotton production. Many measures, including the application of tillage, soil solarisation, soil amendments, and biological controls, are not always effective or efficient to control its damage (Gong et al., 2017; Shan et al., 2018). Thus, it is imperative to search for disease-related genes to breed resistant cultivars. Therefore, in order to promote cotton disease resistance breeding, it is vital to mine novel candidate genes/proteins. In this study, we identified three homologous cotton defence-related genes, *GhWAT1, GhWAT2*, and *GhWAT3*, which possess redundant functions in negatively regulating plant resistance to *V. dahliae*.

The *GhWATs* triple-silenced plants had an increased level of resistance to *V. dahliae*, however, the three single *GhWAT*-silenced plants showed a comparable level of sensitivity to *V. dahliae* with the control plants, suggesting that the three genes redundantly function in plant defence. These results also showed that *GhWATs* in silenced plants acted as negative regulators of plant defence against pathogens. Genes that negatively regulate plant defence can be directly applied through gene mutations. Thus, it is very important to search for negative regulatory defence genes for plant breeding. To date, some negative regulatory genes have been identified in cotton, including *GbJAZ1, GbWRKY1, GhNINJA, Gh14-3-3*, and *GhCYP82D.* The knockout or knockdown of these genes can increase plant resistance against *V. dahliae* infection (Gao et al., 2013; Li et al., 2014; Sun et al., 2014; Wang et al., 2017; Zhang Z.et al., 2018). Therefore, *GhWATs* can be used as candidate genes for cotton resistant cultivar breeding.

GhWATs were located in the tonoplast, and their expression levels were induced by IAA treatment, indicating that *GhWATs* are involved in IAA metabolism and/or IAA transport, which is consistent with results in Arabidopsis (Ranocha et al., 2010, 2013; Denancé et al., 2013). When the *GhWATs* were silenced, auxin transport was blocked, resulting in a local deficiency of auxin in some tissues. Then, that SA synthesis pathway activity was strengthened and promoted SA levels increasing, which increased plant resistance to *V. dahliae* infection. The results showed, there is an inverse correlation between SA and auxin levels in cotton, which has been reported in Arabidopsis (Kazan and Manners, 2009; Ludwig-Muller, 2015; Shigenaga et al., 2017). Additionally, SA biosynthesis via two distinct pathways, ICS catalysed chorismate pathway and PAL mediated synthesis pathway (Wildermuth et al., 2001; Shah, 2003; Vlot et al., 2009). In our studies, both ICS pathway-related genes (*ICS1, EDS1*, and *PAD4*) and PAL pathway-related gene (*PAL*) showed significantly upregulated expression in triple-silenced *GhWATs* plants. Thus the knockdown of *GhWATs* has promoted the accumulation of SA content and increasing the expression level of SA synthesis- and signalling response-related genes, increasing plant defence against pathogens.

The histological analyses of internodes and hypocotyls showed that the knockdown of three *GhWATs* depressed xylem development but increased lignin accumulation in xylem sections. In addition, the three *GhWATs* were predominantly expressed in lignified tissues (roots, internodes, and hypocotyls), indicating that they can involve in tissue lignification. In Arabidopsis, *wat1* mutants showed decreased secondary cell wall thickness in their interfascicular fibres (Ranocha et al., 2010). Similar phenotypes were observed in the *GhWAT123*-silenced plants (Figure 7C). The phenotypes of the *GhWAT123*-silenced showed a decreased xylem area and increased lignin content in the xylem. The lignin synthesis- and lignifications-related genes in the *GhWAT123*-silenced plants were upregulated compared with those in the control, while these silenced-plants showed smaller phenotype, suggesting that knockdown of *GhWATs* increase lignin synthesis and possibly reduces the growth. In Arabidopsis, *wat1* mutants were revealed a defect in stems cell elongation resulting in dwarfed phenotypes (Ranocha et al., 2010). Additionally, XIP1 is an important kinase for stem growth and vascular development, in *xip1* mutant, the highly lignified cell was aberrant accumulation, and defected in oriented cell division, resulting in a dwarfed habit (Bryan et al., 2012). These results imply that *GhWATs* regulate xylem development and lignin accumulation in xylem sections.

Many reports have shown that cotton plant defence against *V. dahliae* involves SA and lignin molecules (Sun et al., 2014; Zhang et al., 2017; Zhu et al., 2018). In the present study, the knockdown of *GhWATs* promoted accumulation of SA content, activated SA pathway-related gene expression and increased lignin accumulation in xylem sections, which facilitated plant resistance to pathogens. Although the G*hWATs*-silenced plants were smaller than the controls, the appropriately small plants possessed higher lodging resistance compared with the large plants, suitable for dense planting and mechanised management. Therefore, *GhWATs* are useful candidate genes for breeding disease resistance cultivars.

## Author Contributions

JW and QP conceived and designed the experiments. YT, ZZ, and YL performed the experiments. JL, GH, MH, and AC constructed the gene editing vectors and data analysis. YT and JW wrote the manuscript. All authors read and approved the final manuscript.

## Conflict of Interest Statement

The authors declare that the research was conducted in the absence of any commercial or financial relationships that could be construed as a potential conflict of interest.

## Footnotes

1 https://www.cottongen.org/icgi/home

2 https://www.uniprot.org/

3 https://blast.ncbi.nlm.nih.gov

4 https://www.cottongen.org/icgi/home

## Supplementary Material

FIGURE S1 An amino acid alignment of WAT proteins from *G. hirsutum, A. thaliana*, and *Medicago sativa*. The complete amino acid sequences of five WATs were retrieved from NCBI database and aligned by DNAMAN 7.0. The two DUF6 domains were noted with black underlines. Go, *Gossypium hirsutum*; Mt, *Medicago sativa*; At, *Arabidopsis thaliana*.

FIGURE S2 The tissue expression patterns of three *GhWATs*. Expression levels of three *GhWATs* in various tissues. Roots, internodes, leaves, cotyledons, and hypocotyls were sampled from 3-week-old seedlings. *GhUB-7* was used as the reference gene. The error bars represent the standard error of the mean (SEM) of three biological replicates. More than three individual samples were collected on each of the biological replicates.

FIGURE S3 The detection of three *GhWATs* genes expression levels in *GhWAT1*-, *GhWAT2*-, *GhWAT3*-, *GhWAT123*-silenced, and the control plants. **(A,B)** The relative expression levels of *GhWAT1, GhWAT2*, and *GhWAT3* were tested in the leaf **(A)** and root **(B)** of the corresponding *GhWAT1*-, *GhWAT2*-, *GhWAT3*-silenced plants compared with the control plans, respectively. **(C)** The relative expression levels of *GhWAT1, GhWAT2*, and *GhWAT3* were tested in the leaves and roots of the *GhWAT123*-silenced plants compared with the control plants. TRV:00 is control plants injected with the pYL156 vector, which relative expression levels were normalised to ‘1’. *GhUB-7* was used as the reference gene. Error bars represent the SEM of three technical replicates. Student’s *t*-test was used to determine significant differences between corresponding TRV:00 and TRV:GhWAT123 plants (^∗∗^*P* < 0.01, ^∗∗∗^*P* < 0.001).

FIGURE S4 The phenotypes and growth of *GhWATs*-silencing plants. **(A)** *GhWAT1*-, *GhWAT2*-, and *GhWAT3*-silenced plants showed comparable phenotypes with the control, while *GhWAT123-*silenced plants exhibited clearly shorter in height than the control at 21 days post-Agro-infiltration. **(B)** The fresh weight of the control and silenced plants, TRV:00 is control plants injected with the pYL156 vector, Student’s *t*-test was used to determine significant differences between the control and silenced plants. The error bars represent the standard error of the mean (SEM) of three biological replicates. Each time was collected more than five seedlings (^∗^*P* < 0.05).

FIGURE S5 The lignin content and the expression levels of lignifications- and lignin synthesis-related genes in *GhWAT123*-silenced and the control plants. **(A)** The contents of acid-insoluble lignin residues in hypocotyls of *GhWAT123*-silenced and the control plants by Klason method. The same experiments were performed three times; each time was measured more than five seedlings. **(B)** The expression levels of lignifications-related genes *GhLac1, Ghpox1, GhDIR1* in triple-silenced plants and the control. **(C)** The relative expression levels of lignin synthesis-related genes in *GhWAT123*-silenced and the control plants. *GhUB-7* was used as the reference gene. The relative expression levels analyses were normalised by control treated data. TRV:00 is control plants injected with the pYL156 vector. The Student’s *t*-test was performed on statistically significant differences analysis (^∗^*P* < 0.05, ^∗∗^*P* < 0.01). Error bars represent the SEM of three biological replicates; each time was collected three seedlings.

TABLE S1 Primer table.
