# Supplementary figures and images for: Cotton WATs Modulate SA Biosynthesis and Local Lignin Deposition Participating in Plant Resistance Against Verticillium dahliae
Source: Front Plant Sci. 2019 Apr 26;10:526. doi: 10.3389/fpls.2019.00526 (PMC6499033; doi:10.3389/fpls.2019.00526)

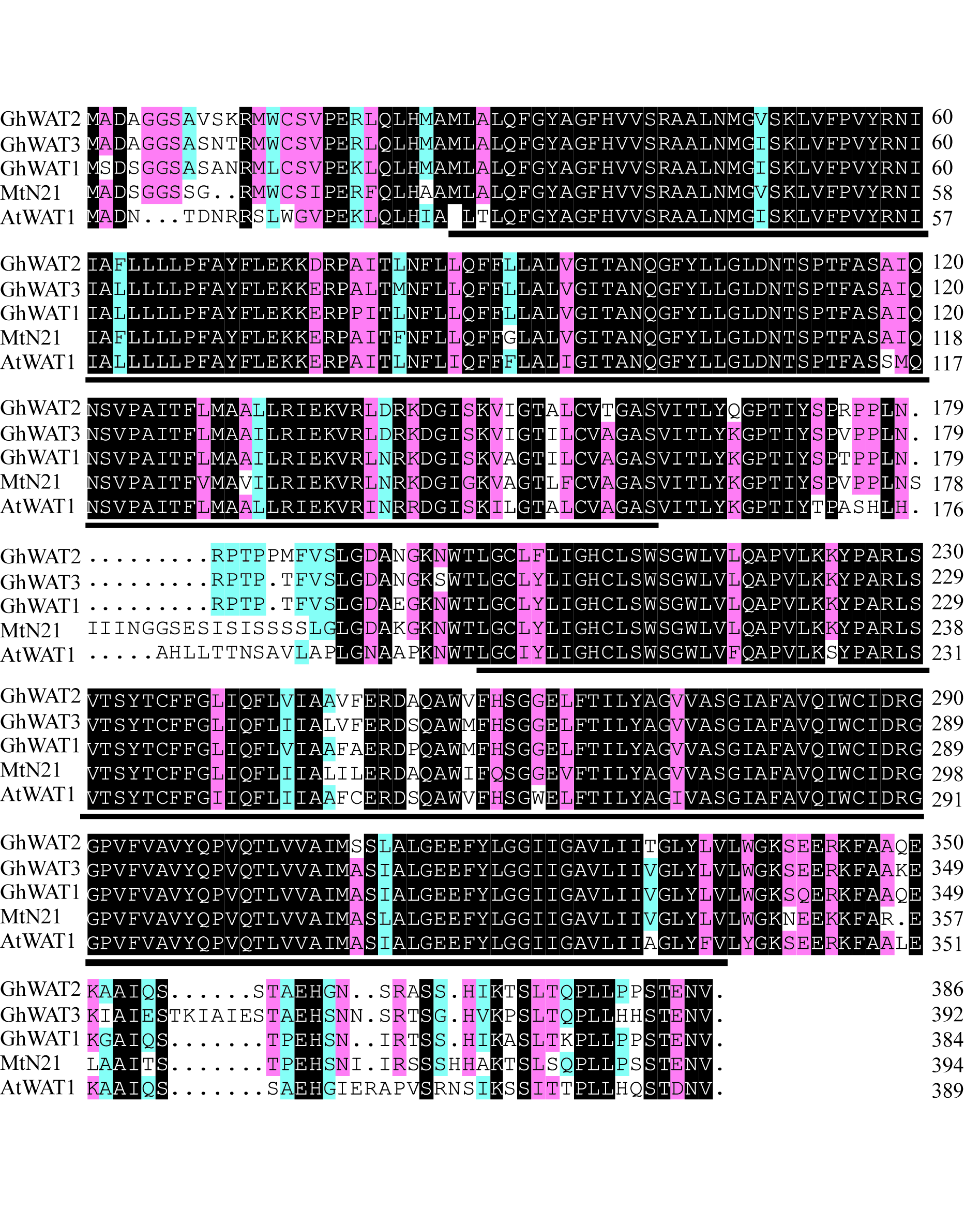

Supplement: FIGURE S1 — An amino acid alignment of WAT proteins from G. hirsutum, A. thaliana, and Medicago sativa. The complete amino acid sequences of five WATs were retrieved from NCBI database and aligned by DNAMAN 7.0. The two DUF6 domains were noted with black underlines. Go, Gossypium hirsutum; Mt, Medicago sativa; At, Arabidopsis thaliana. [file Image_1.TIF]

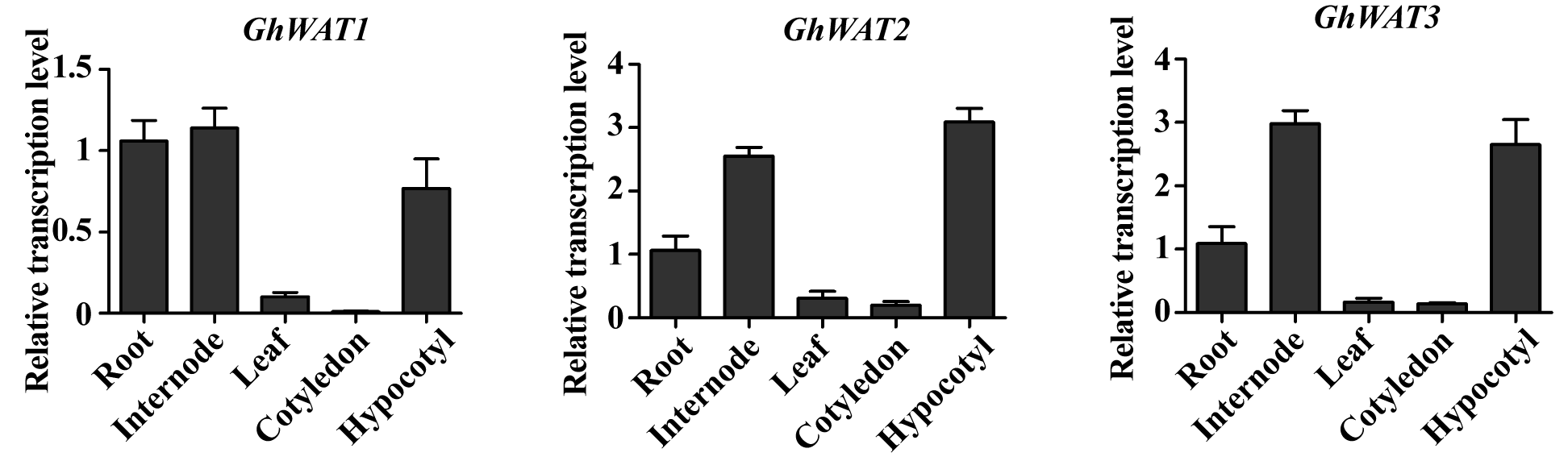

Supplement: FIGURE S2 — The tissue expression patterns of three GhWATs. Expression levels of three GhWATs in various tissues. Roots, internodes, leaves, cotyledons, and hypocotyls were sampled from 3-week-old seedlings. GhUB-7 was used as the reference gene. The error bars represent the standard error of the mean (SEM) of three biological replicates. More than three individual samples were collected on each of the biological replicates. [file Image_2.TIF]

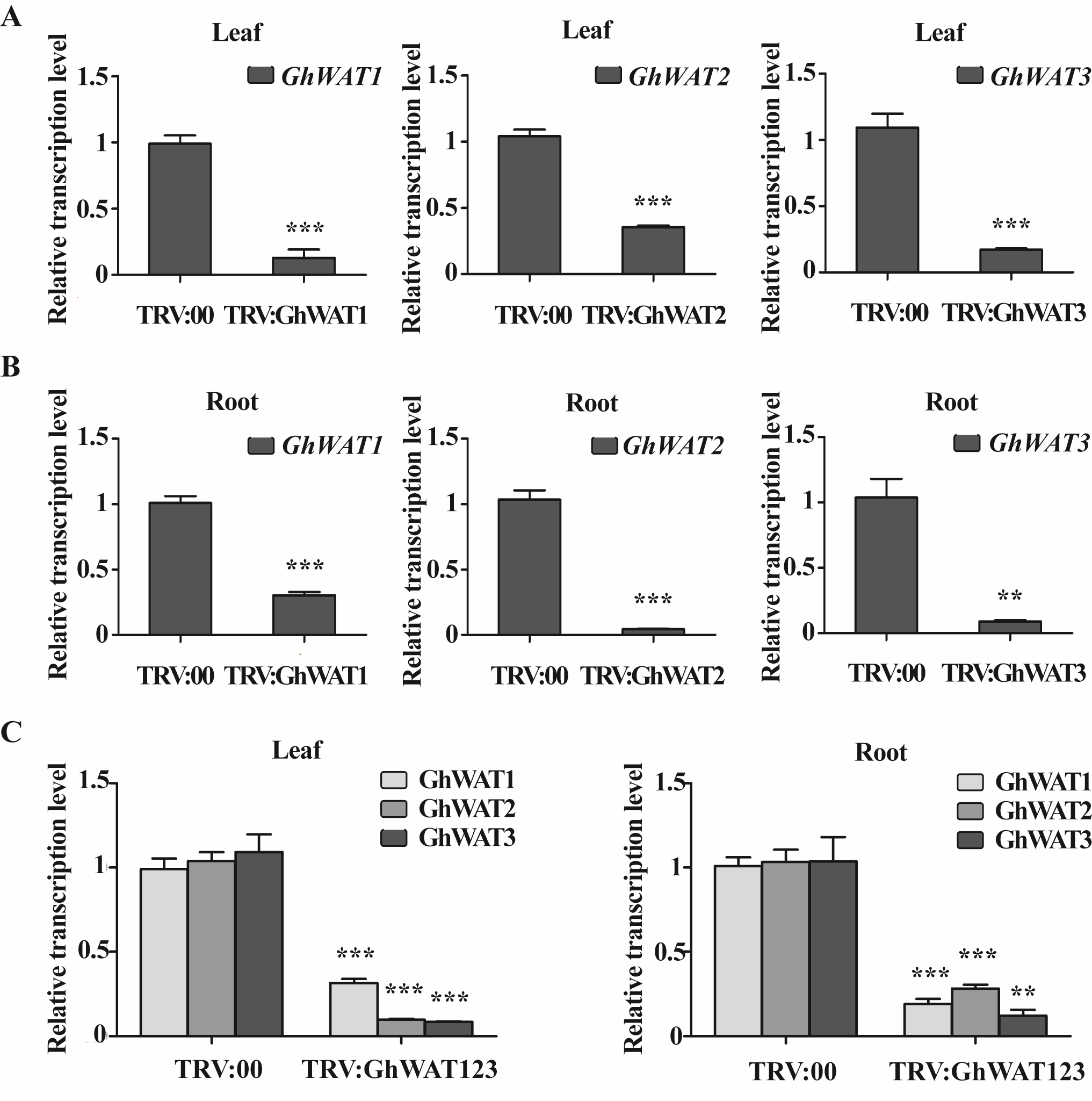

Supplement: FIGURE S3 — The detection of three GhWATs genes expression levels in GhWAT1-, GhWAT2-, GhWAT3-, GhWAT123-silenced, and the control plants. (A,B) The relative expression levels of GhWAT1, GhWAT2, and GhWAT3 were tested in the leaf (A) and root (B) of the corresponding GhWAT1-, GhWAT2-, GhWAT3-silenced plants compared with the control plans, respectively. (C) The relative expression levels of GhWAT1, GhWAT2, and GhWAT3 were tested in the leaves and roots of the GhWAT123-silenced plants compared with the control plants. TRV:00 is control plants injected with the pYL156 vector, which relative expression levels were normalised to ‘1’. GhUB-7 was used as the reference gene. Error bars represent the SEM of three technical replicates. Student’s t-test was used to determine significant differences between corresponding TRV:00 and TRV:GhWAT123 plants (∗∗P < 0.01, ∗∗∗P < 0.001). [file Image_3.TIF]

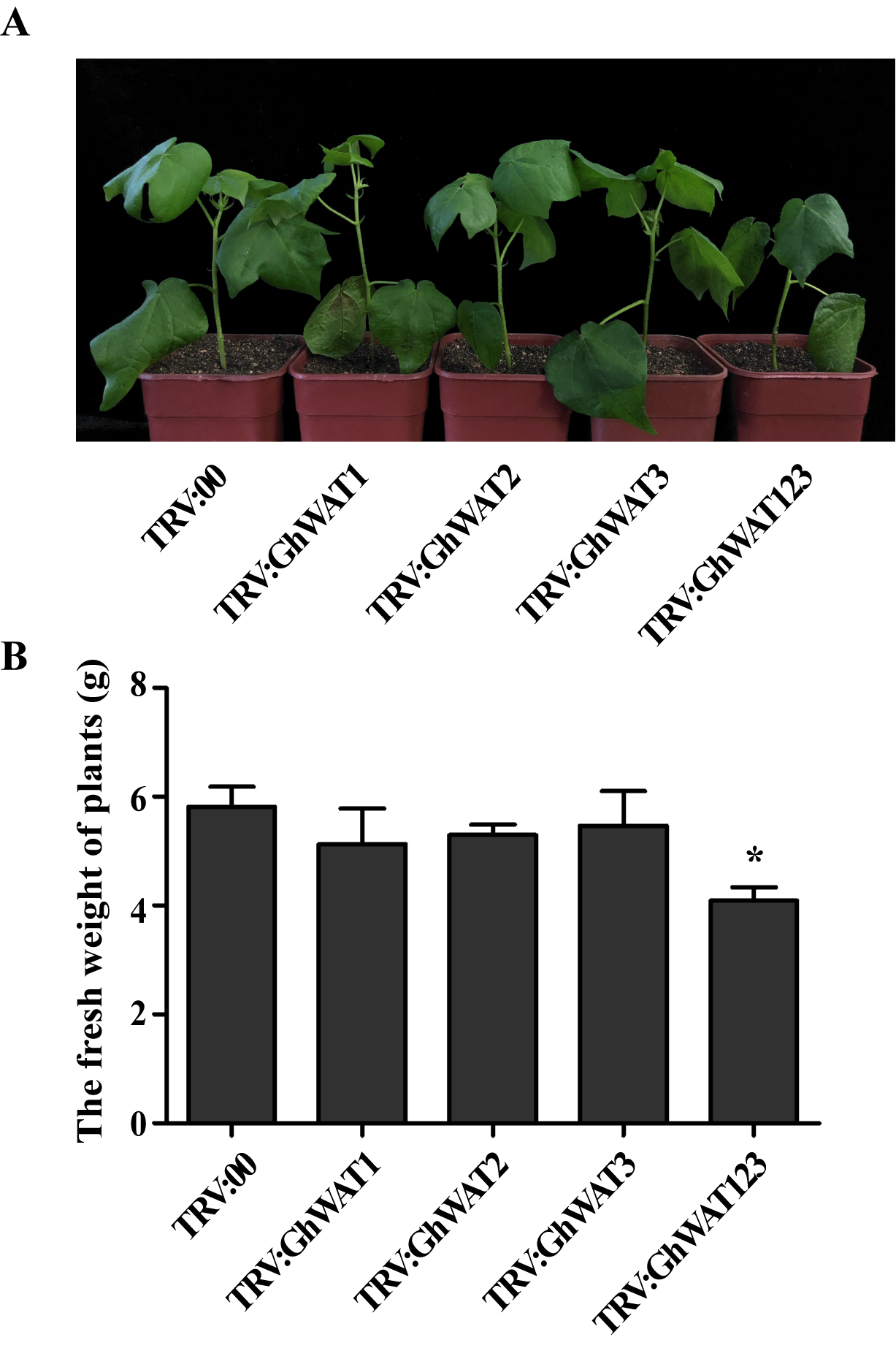

Supplement: FIGURE S4 — The phenotypes and growth of GhWATs-silencing plants. (A) GhWAT1-, GhWAT2-, and GhWAT3-silenced plants showed comparable phenotypes with the control, while GhWAT123-silenced plants exhibited clearly shorter in height than the control at 21 days post-Agro-infiltration. (B) The fresh weight of the control and silenced plants, TRV:00 is control plants injected with the pYL156 vector, Student’s t-test was used to determine significant differences between the control and silenced plants. The error bars represent the standard error of the mean (SEM) of three biological replicates. Each time was collected more than five seedlings (∗P < 0.05). [file Image_4.TIF]

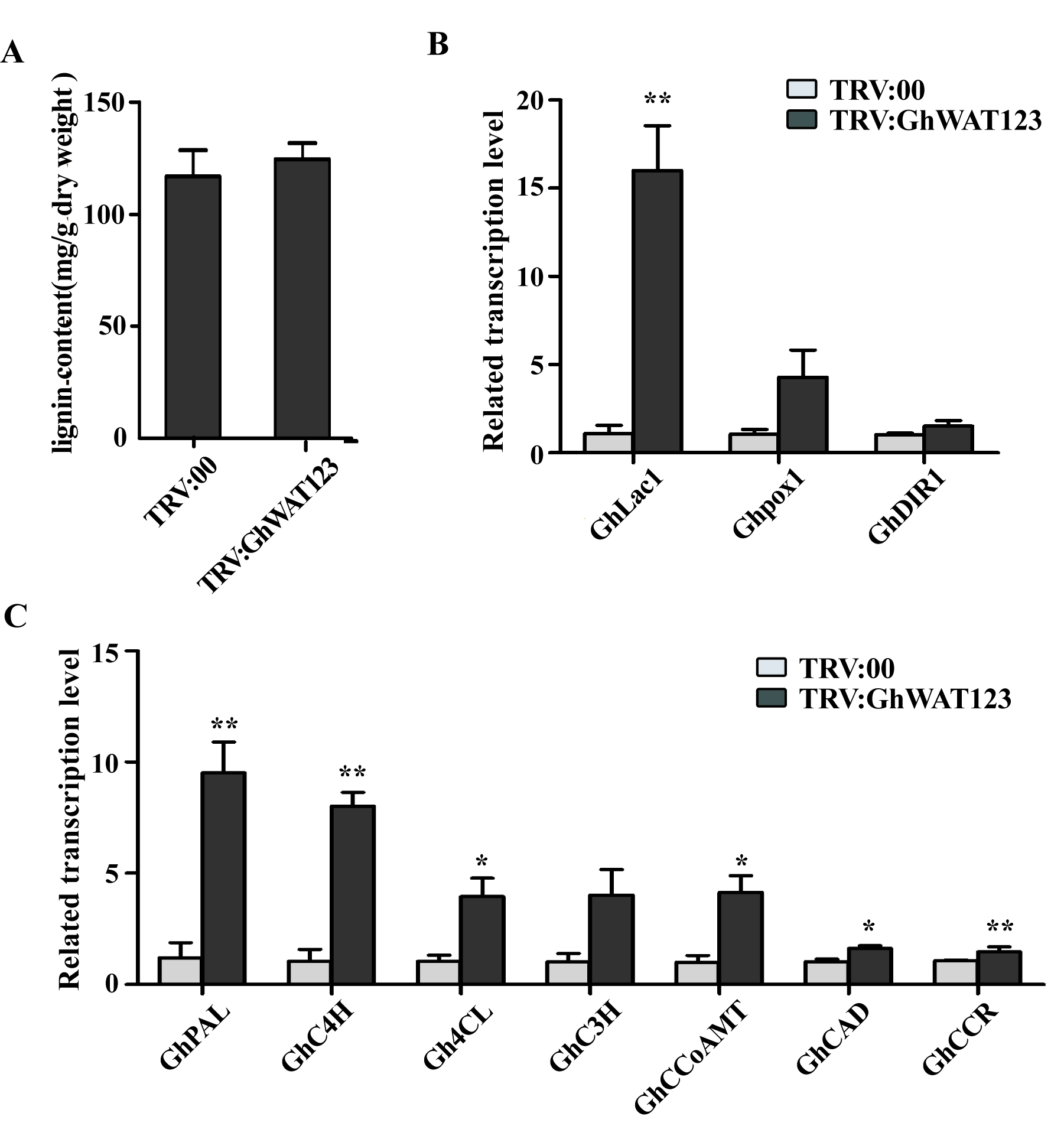

Supplement: FIGURE S5 — The lignin content and the expression levels of lignifications- and lignin synthesis-related genes in GhWAT123-silenced and the control plants. (A) The contents of acid-insoluble lignin residues in hypocotyls of GhWAT123-silenced and the control plants by Klason method. The same experiments were performed three times; each time was measured more than five seedlings. (B) The expression levels of lignifications-related genes GhLac1, Ghpox1, GhDIR1 in triple-silenced plants and the control. (C) The relative expression levels of lignin synthesis-related genes in GhWAT123-silenced and the control plants. GhUB-7 was used as the reference gene. The relative expression levels analyses were normalised by control treated data. TRV:00 is control plants injected with the pYL156 vector. The Student’s t-test was performed on statistically significant differences analysis (∗P < 0.05, ∗∗P < 0.01). Error bars represent the SEM of three biological replicates; each time was collected three seedlings. [file Image_5.TIF]
